# The Transcriptional Co-Regulator HCF-1 Is Required for INS-1 β-cell Glucose-Stimulated Insulin Secretion

**DOI:** 10.1371/journal.pone.0078841

**Published:** 2013-11-08

**Authors:** Terri N. Iwata, Timothy J. Cowley, Michael Sloma, Yewei Ji, Hana Kim, Ling Qi, Siu Sylvia Lee

**Affiliations:** Department of Molecular Biology and Genetics, Cornell University, Ithaca, New York, United States of America; GDC, Germany

## Abstract

The transcriptional co-regulator host cell factor-1 (HCF-1) plays critical roles in promoting cell cycle progression in diverse cell types, and in maintaining self-renewal of embryonic stem cells, but its role in pancreatic β-cell function has not been investigated. Immunhistochemistry of mouse pancreas revealed nuclear expression of HCF-1 in pancreatic islets. Reducing HCF-1 expression in the INS-1 pancreatic β-cell line resulted in reduced cell proliferation, reduced glucose-stimulated insulin secretion, and reduced expression of the critical β-cell transcription factor Pdx1. HCF-1 is a known co-activator of the E2F1 transcription factor, and loss of E2F1 results in pancreatic β-cell dysfunction and reduced expression of Pdx1. Therefore we wondered whether HCF-1 might be required for E2F1 regulation of Pdx1. Chromatin immunoprecipitation experiments revealed that HCF-1 and E2F1 co-localize to the *Pdx1* promoter. These results indicate that HCF-1 represents a novel transcriptional regulator required for maintaining pancreatic β-cell function.

## Introduction

Diabetes develops due to a deficiency in circulating insulin caused by pancreatic β-cell destruction and/or impaired β-cell function. In type 1 diabetes, pancreatic β-cells are selectively destroyed resulting in reduced β-cell mass, while in type 2 diabetes, loss of insulin-secretory ability as well as β-cell apoptosis lead to defects in glucose homeostasis [1], [2]. Understanding the factors responsible for maintaining β-cell mass and β-cell function is, therefore, a key step in developing therapeutics to prevent the development of diabetes.

While a number of key DNA-binding transcription factors are known to be critical in regulating the proliferation, survival, differentiation, and proper functioning of β-cells [3], [4], relatively little is known about the transcriptional co-factors that act to assemble appropriate transcriptional complexes and enable transcription factors to carry out their functions. The transcriptional co-regulator host cell factor-1 (HCF-1) is emerging as a critical co-factor to many different DNA-binding transcription factors with key roles ranging from cell cycle progression [5], [6] and DNA-damage induced apoptosis [7] to maintenance of embryonic stem cell pluripotency [8]. HCF-1 contains multiple protein-protein interaction domains [9] but has no detectable DNA-binding or enzymatic activity. Instead, HCF-1 largely functions as a scaffolding protein assembling appropriate transcriptional complexes at target gene promoters, and bridging interactions between transcription factors and chromatin remodeling factors [7], [10]–[12]. Given HCF-1’s ability to associate with, and modulate the function of, a variety of transcription factors including the cell cycle regulating E2F family proteins [12], the embryonic stem cell pluripotency factor Ronin [8], the Schwann cell differentiation factor Krox20 [13], and metabolic and stress-regulating proteins such as PGC-1a [14] and FoxO [15], we hypothesized that HCF-1 will also play a key role in pancreatic β-cell function. In this study, we demonstrate an essential role for HCF-1 in glucose-stimulated insulin secretion in the INS-1 pancreatic β-cell line suggesting that HCF-1 represents a promising future therapeutic target for the prevention and treatment of diabetes.

## Materials and Methods

### Ethics Statement

All animal procedures were approved by the Cornell University Institutional Animal Care and Use Committee (#2007-0051).

### Immunohistochemistry (IHC)

6-week-old male C57BL/6 mice were fed with 60% HFD for 12 weeks. Pancreas tissues were collected from these mice and fixed in 10% formaldehyde, and processed by the Cornell Histology Core Facility for sectioning. Paraffin-embedded pancreas tissue were rehydrated, boiled in 1 mM EDTA for antigen retrieval, and stained with Histostain kit and DAB substrate from Invitrogen. Primary antibodies used for immunohistochemistry were 1∶100 dilution of HCF-1 (Bethyl Labs) or 1∶100 dilution of anti-rabbit-IgG (Santa Cruz). IHC sections were scanned using the Aperio Scanscope.

### Cell Culture, siRNA Transfection, and shRNA Cell Selection and Induction

The INS-1 rat insulinoma cell line was a gift from L. Qi [16] and was maintained and transfected with siRNA as previously described, with some modifications [15]. In brief, INS-1 cells were transfected twice, one day apart, with 5 nM siRNA using Lipofectamine RNAiMax per the manufacturer’s protocol. siRNA duplexes directed against rat *HCF-1* were purchased from Dharmacon and targeted the following sequences:; siHCF-1 #1∶5′-AGAACAACATTCCGAGGTA-3′; siHCF-1 #2∶5′-GCTTATAAATTTCGAGTTG-3; siHCF-1 #3 5′-CGGCAAGATTATCGAGTAC-3′; siHCF-1 #4∶5′-GGAAGAGACTGAAGGCAAA-3′. Non-targeting control siRNA was also from Dharmacon.

Inducible shRNA-mediated *HCF-1* knockdown INS-1 cells were generated by transduction with a pInducer10 lentiviral construct expressing the shRNA from a tetracycline sensitive promoter [17]. The *HCF-1* shRNA construct targets the following sequence: shHCF1∶5′-CCCGAGGTACCTGAATGACTTA-3′. shRNA construct targeting luciferase (shLuc) was used as a control. The shLuc target sequence is as follows: shLuc: 5′-ACTGAAGTCTCTGATTAAGTAC-3′. VSV-G pseudotyped lentiviral particles were generated by calcium phosphate transfection of 293T cells with the pInducer10-shRNA construct, psPAX2 packaging plasmid, and pMD2.G VSV-G plasmid. Cells were selected and maintained in media containing 0.6 µg/mL puromycin. Experiments were carried out in the absence of puromycin. shRNA was induced by adding doxycycline to the media at a concentration of 4 µg/mL. Induction was maintained throughout the course of experiments.

### Immunoblotting

Preparation of cell lysates and immunoblotting were performed as described previously [15]. The following antibodies were used: HCF-1 (Bethyl Labs), β-actin (Millipore), Pdx1 (Cell signaling). Densitometric analysis was performed using ImageJ software.

### Cell Growth Curve and BrdU Incorporation Assays

For siRNA cell growth curve experiments, 10^5^ cells were seeded in each well of a 6-well plate at the time of the first siRNA transfection. 3 wells per condition were counted using the trypan blue exclusion method. For shRNA experiments, 2.5×10^4^ cells were seeded in each well of a 6-well plate and induced with doxycycline. 3 wells per condition were counted using the trypan blue exclusion method.

For siRNA BrdU incorporation experiments, 10^5^ cells were seeded in each well of a 6-well plate containing a glass coverslip at the time of the first siRNA transfection. Three days after the first siRNA transfection, cells were incubated in media containing 10 µM BrdU (Sigma). The following day, cells were fixed and stained with anti-HCF-1 and anti-BrdU antibody [6]. Cells were fixed in 2% paraformaldehyde, permeabilized with 0.5% TritonX-100, and blocked for 20 minutes in 3% BSA. Coverslips were incubated with anti-HCF-1 antibody (Bethyl Labs) for 1 hour, washed and incubated with cy3-conjugated anti-rabbit IgG for 30 minutes, fixed and DNA denatured with 4N HCl for 20 minutes, and incubated with FITC-conjugated anti-BrdU (BD Biosciences) antibody. Nuclei were stained with DAPI. Coverslips were mounted on glass slides using Vectashield (Vector Labs). Coverslips were examined and pictures obtained on a Leica DM 5000B fluorescent microscope. At least 400 cells were counted per siRNA treatment.

For shRNA BrdU incorporation experiments, cells were seeded on a 10 cm dish such that approximately 2×10^6^ cells would be present at the time of the assay. Cells were induced to express shRNA for 2, 4, or 6 days with doxycycline treatment, and were then incubated with 10–20 µM BrdU for 30 minutes. Immediately following BrdU incubation, cells were trypsinized and washed in 1%BSA, fixed in 70% ethanol and kept at −20°C until all samples were collected. 5×10^5^ fixed cells were denatured with 2N HCl/0.5% Triton X-100 for 30 minutes, neutralized with 0.1 M sodium tetraborate, pH 8.5, resuspended in 0.5 mL 0.5% Tween 20/1% BSA/PBS, then incubated with FITC-conjugated anti-BrdU (BD Biosciences) antibody. Cells were washed with 0.5% Tween 20/1%BSA/PBS and counted on an LSR II (Becton Dickinson) flow cytometer.

### Reverse-transcription Coupled Quantitative PCR (RT-qPCR)

RNA was isolated from INS-1 cells treated with the indicated siRNAs using Trizol reagent and was reverse-transcribed using Superscript III First-Strand kit (Invitrogen). cDNAs were analyzed by quantitative-PCR using the SYBR Green system on a Roche LightCycler 480 real time PCR machine and quantified relative to a standard curve. *β-actin* was used as an internal control. Primer sequences can be found in Table S1.

### Glucose-stimulated Insulin Secretion and Intracellular Insulin Content Analysis

For siRNA experiments, 10^6^ INS-1 cells were sequentially transfected with sicontrol or *HCF-1* siRNA in 10 cm plates. One day after the second siRNA transfection, cells were trypsinized and seeded at 10^6^ cells per well in 24-well plates in RPMI medium containing 5 mM glucose. The following day, cells were washed once with warm KRB buffer (119 mM NaCl, 4.74 mM KCl, 2.54 mM CaCl_2_, 1.19 mM MgSO_4_, 1.19 mM KH_2_PO_4_, 25 mM NaHCO_3,_ 10 mM HEPES and 0.2% fatty acid free BSA) and incubated for one hour in 0.5 mL KRB buffer at 37°C. Cells were then incubated in 0.5 mL of KRB buffer containing either 3 mM or 16.7 mM glucose for 1 hour at 37°C. Supernatant was collected and insulin measured using a rat/mouse insulin ELISA kit (Millipore). Cells were lysed in cell lysis buffer or incubated with acidified ethanol (75% ethanol, 1.5% HCl) overnight at −20°C to measure intracellular insulin content. Insulin measurements were normalized to cellular protein content as determined by Bradford assay. For shRNA experiments, INS-1 cells were treated with 6 days of doxycycline induction. Insulin secretion assay and ELISA were performed as described above.

### Chromatin Immunoprecipitation (ChIP) Assays

1.5×10^6^ INS-1 cells were transfected with siRNA in 10 cm plates as described above. Two days after the second siRNA transfection, cells were cross-linked with 1% formaldehyde for 10 minutes at room temperature. Glycine was added to a final concentration of 125 mM for 5 minutes. Cells were washed with cold PBS and lysed in buffer containing 25 mM HEPES, 1.5 mM MgCl2, 10 mM KCl, 0.5% NP40, 1 mM DTT and protease inhibitors. Nuclei were pelleted and lysed in buffer containing 50 mM HEPES, 140 mM NaCl, 1 mM EDTA, 1% Triton X-100, 0.1% sodium deoxycholate, and protease inhibitors, and sonicated using a Diogenode Bioruptor. Chromatin was immunoprecipitated overnight using antibodies against HCF-1 (Bethyl labs A301-399A) or E2F1 (Santa Cruz, C-20) and recovered with protein A agarose resin (Thermo Scientific). The protein A resin was washed, immunoprecipitated complexes eluted, and crosslinks reversed. DNA was purified using a Qiagen PCR purification kit, and assayed by quantitative PCR. Primer sequences can be found in Table S1.

### Data Analysis

Data are represented as mean +/− SEM and analyzed by the Student’s t-test, except for the BrdU incorporation assay, which is represented as mean and 95% confidence interval and analyzed by Pearson’s Chi-squared test with Yates’ correction. A p-value of less than 0.05 was considered to be statistically significant.

## Results and Discussion

**Figure 1:**
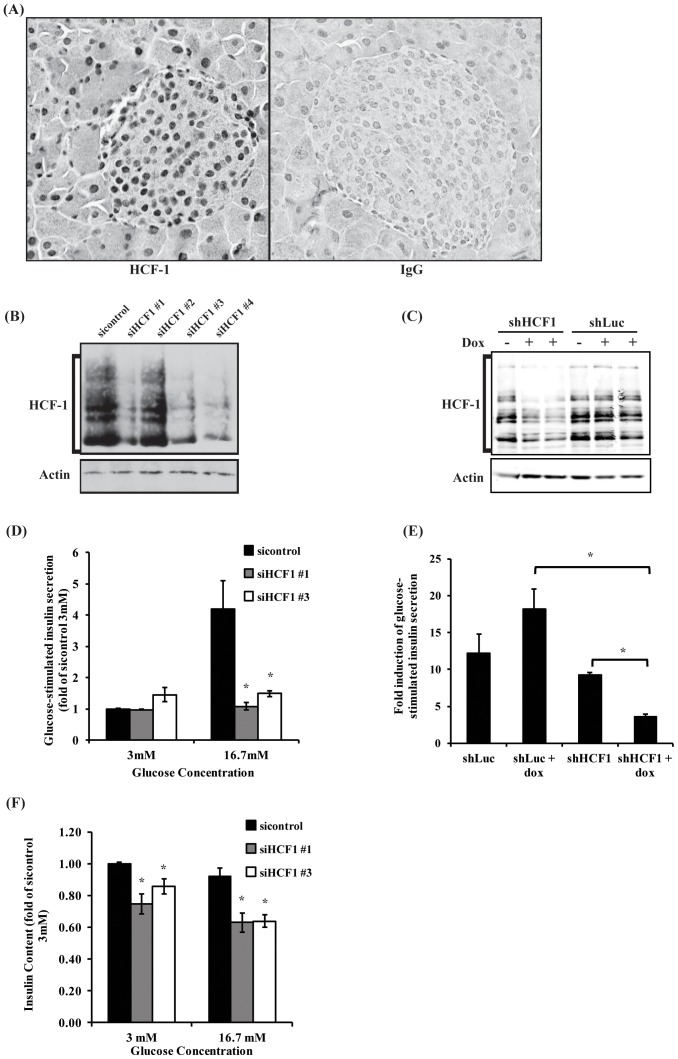
HCF-1 is required for glucose-stimulated insulin secretion. (A) Immunohistochemistry showing HCF-1 expression in mouse pancreas. Pancreas from 6-week-old male C57BL/6 mice was stained with HCF-1 (left panel) or IgG-control (right panel). HCF-1 is expressed in the nuclei of cells from the exocrine pancreas and the islet of Langerhans. (B) Western blot analysis of HCF-1 protein levels from INS-1 cells transfected twice with control or four different *HCF-1* siRNAs. (C) Western blot analysis of HCF-1 levels from cells induced to express shLuc or shHCF-1 shRNA with doxycycline treatment. (D) Glucose-stimulated insulin secretion analysis of cells treated with *HCF-1* siRNA. Insulin secretion determined by insulin ELISA and normalized to total cellular protein. Results shown are pooled from three independent experiments, and represent mean +/− SEM. *denotes a p-value <0.05 relative to sicontrol (Student’s t-test). (E) Glucose-stimulated insulin secretion of INS-1 cells expressing shLuc or shHCF1 shRNA. Fold induction calculated as the ratio of insulin secretion from cells treated with 16.7 mM glucose relative to cells treated with 3 mM glucose. Data shown are from three independent experiments, and are expressed as mean +/− SEM. *denotes a p-value <0.05 (Student’s t-test). (F) Cellular insulin content analysis of cells treated with *HCF-1* siRNA. Insulin content determined by insulin ELISA and normalized to total cellular protein. Results shown are pooled from three independent experiments, and represent mean +/− SEM. *denotes a p-value <0.05 relative to sicontrol (Student’s t-test).

To probe a possible function of HCF-1 in pancreatic β-cells, we first examined the expression of HCF-1 in mouse pancreas by immunohistochemistry (Figure 1A). We found that HCF-1 is broadly expressed within the pancreas with both exocrine pancreas and islet of Langerhans cells exhibiting strong nuclear HCF-1 expression.

To characterize the role of HCF-1 in pancreatic β-cells, we utilized HCF-1 siRNA to deplete HCF-1 in the INS-1 pancreatic β-cell line (Figure 1B). Consistent with previous studies showing the importance of HCF-1 in regulating cell cycle progression in various cell lines [5], [6], [12], INS-1 β-cells treated with three different *HCF-1* targeting siRNAs exhibited defects in cell growth over time as well as decreased BrdU incorporation (Figure S1A and S1C) indicating a defect in cell proliferation. To further confirm the observations with siRNA-mediated knockdown of HCF-1, we engineered an inducible shRNA lentivirus targeting *HCF-1* mRNA and generated a stable INS-1 cell line which conditionally expressed this shRNA upon treatment with doxycycline (Figure 1C). Similar to our observations with the *HCF-1* siRNA-treated cells, INS-1 cells with shRNA-mediated knockdown of HCF-1 also showed decreased cell growth and proliferation over time (Figure S1B and S1D).

We next examined whether HCF-1 also affects these cells’ functional ability to secrete insulin in response to glucose. INS-1 β-cells transfected with control siRNA exhibited a robust 4-fold increase in insulin secretion when stimulated with high (16.7 mM) vs low (3 mM) glucose, as determined by ELISA analysis (Figure 1D). *HCF-1* siRNA-treated cells (si#1 and si#3), by contrast, did not show any increase in insulin secretion in response to high glucose, indicating that HCF-1 is absolutely required for glucose-stimulated insulin secretion in the INS-1 β-cell model. shRNA-mediated knockdown of *HCF-1* similarly led to a significant decrease in glucose-stimulated insulin secretion (Figure 1E), albeit not as robust as observed with siRNA-mediated knockdown of *HCF-1* which is likely due to differences in knockdown levels achieved with siRNA versus shRNA (compare Figure 1B to Figure 1C). We then examined whether the reduced insulin secretion phenotype might arise from an overall reduction in insulin content in these cells. Indeed, analysis of the intracellular insulin content showed that HCF-1 knockdown cells have reduced levels of insulin (Figure 1F), indicating that the decrease in the cellular insulin pool may contribute to the reduction in glucose-stimulated insulin secretion. However, as the reduction in intracellular insulin levels are modest, and the amount of insulin secreted represents a small fraction (less than 1/10^th^) of the total intracellular insulin available, additional defects associated with insulin secretion likely represent the major contributor to the greatly impaired ability of the HCF-1 knockdown cells to secrete insulin in response to high glucose.

**Figure 2:**
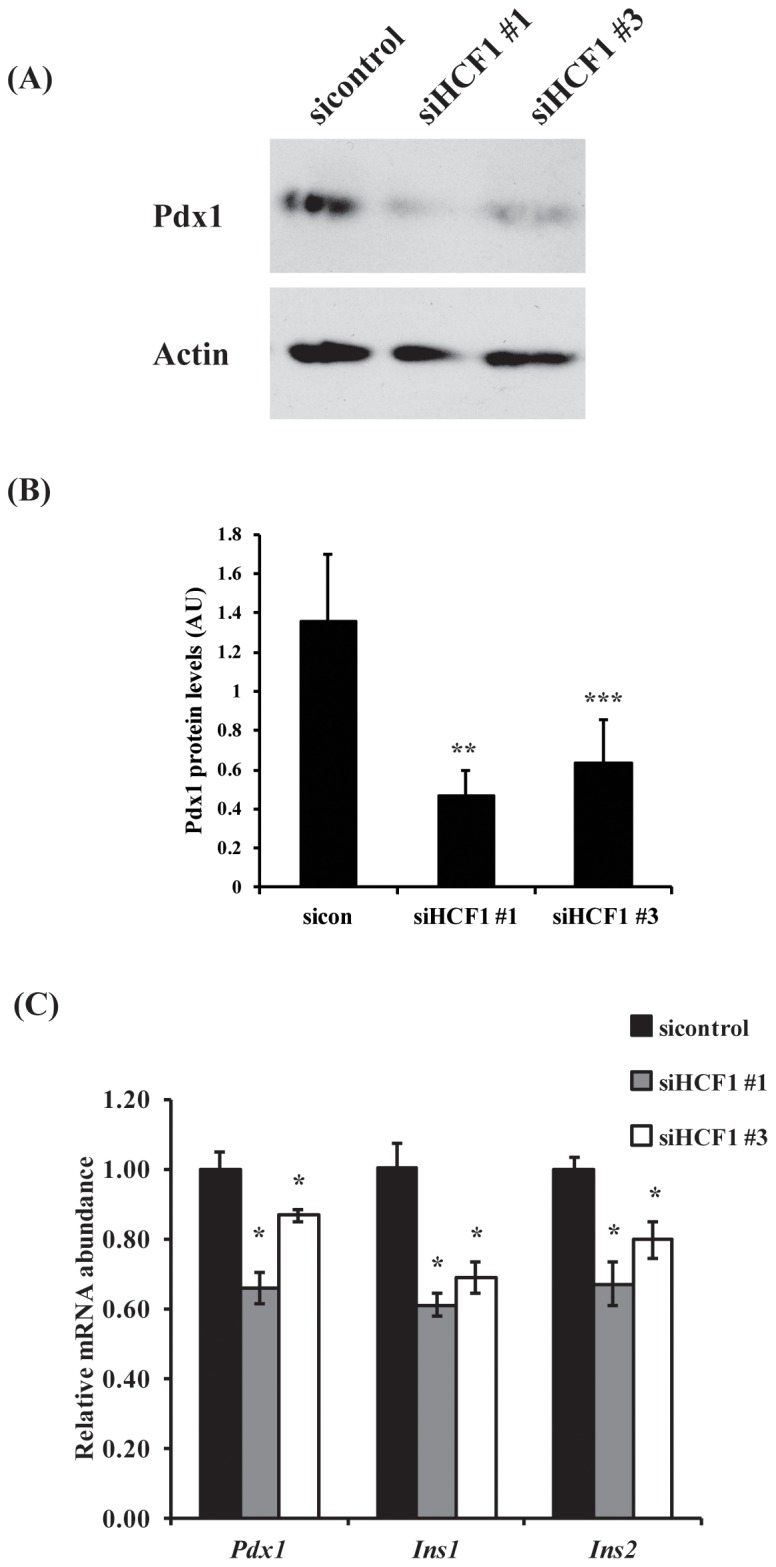
HCF-1 regulates expression of Pdx-1. (A) Western blot analysis of Pdx-1 protein levels in HCF-1 knockdown cells. (B) Relative Pdx1 protein levels as determined by densitometry analysis. Pdx1 levels were normalized to β-actin levels. Results are pooled from 5 experiments and represent mean +/− SEM. **denotes a p-value = 0.03, ***denotes a p-value = 0.05 (Student’s t-test). (C) RT-qPCR analysis of *Pdx1*, *Ins1* and *Ins2* transcript levels in HCF-1 knockdown cells. The mean normalized RNA level for each gene in sicontrol treated cells was set to 1. The data shown are pooled from three independent experiments and are represented as mean +/− SEM. *denotes a p-value <0.05 relative to cells treated with sicontrol siRNA (Student’s t-test).

In mature β-cells, the transcription factor pancreatic duodenal homeobox 1 (Pdx1) promotes insulin gene transcription and insulin secretion [18], [19] and is required for β-cell proliferation [20]. Decreased *Pdx1* expression results in reduced cellular insulin content and reduced glucose-stimulated insulin secretion [21], which are phenotypes we observe with reduced HCF-1 expression. We therefore wondered whether HCF-1 may in fact regulate the expression of Pdx1. Indeed, we found that *HCF-1* siRNA treated cells showed significant reductions in levels of Pdx1 protein (Figure 2A and 2B) and mRNA transcripts (Figure 2C). The Pdx1 target genes *Ins1* and *Ins2* also showed significantly reduced expression with HCF-1 knockdown (Figure 2C), correlating well with our observation of reduced intracellular insulin levels in cells depleted of HCF-1. These results suggest that loss of HCF-1 leads to diminished Pdx1 activity through reductions in Pdx1 expression, which likely contribute to reduce proliferation and insulin secretion in β-cells. Importantly, human *PDX1* mutations are associated with the development of diabetes [22]–[24]. Thus, as a modulator of Pdx1 expression, HCF-1 represents a novel β-cell factor implicated in affecting diabetes development and progression.

We reasoned that HCF-1, as a known transcriptional co-regulator, likely regulates *Pdx1* gene expression by modulating the activity of a DNA-binding transcription factor which itself acts upon the *Pdx1* promoter. Among the known HCF-1 transcription factor partners, FoxO1 and E2F1 have been implicated in pancreatic β-cell regulation. In β-cells, FoxO1 inhibits the expression of the *Pdx1* gene by opposing FoxA2-mediated transcription of the *Pdx1* gene [25]. FoxO1 also represses Pdx1 transcriptional activity by affecting Pdx1 nuclear translocation [25], [26]. Recently, HCF-1 was implicated to function as a novel repressor of FoxO transcription factors in mammals [15]. Furthermore, in *C. elegans*, HCF-1 represses the transcriptional activity of the *C*. *elegans* FoxO homolog by binding to it, thus preventing it from localizing to the promoters of its target genes [27].

In contrast to a role for HCF-1 as a FoxO1 repressor, HCF-1 is a known co-activator of the E2F1 transcription factor and functions to bridge interactions between E2F1 and chromatin modifying enzyme complexes at the gene promoter [12]. Like FoxO1, E2F1 has also been shown to function as a critical pancreatic β-cell transcription factor. E2F1 null mice have reduced expression of *Pdx1*, reduced pancreatic β-cell mass and reduced pancreatic insulin content [28]. E2F1 also regulates insulin secretion through promoting the expression of the K_ATP_ channel, *Kir6.2*, involved in glucose-stimulated insulin secretion [29].

FoxO1 has been shown to bind to the consensus forkhead binding sequence located in the highly conserved Pdx1 homology 2 (PH2) region of the mouse *Pdx1* promoter [25]. Examination of the proximal promoter region of the rat *Pdx1* promoter revealed an additional consensus forkhead binding sequence located 186 bp upstream of the transcription start site. We performed chromatin immunoprecipitation (ChIP) to determine whether FoxO1 binding to the *Pdx1* promoter may be affected by HCF-1 as would be predicted based on a previously proposed model of HCF-1-mediated repression of FoxO activity [27]. However, we were unable to detect a significant enrichment of FoxO1 at the *Pdx1* promoter with or without *HCF-1* knockdown under our experimental conditions (data not shown).

**Figure 3:**
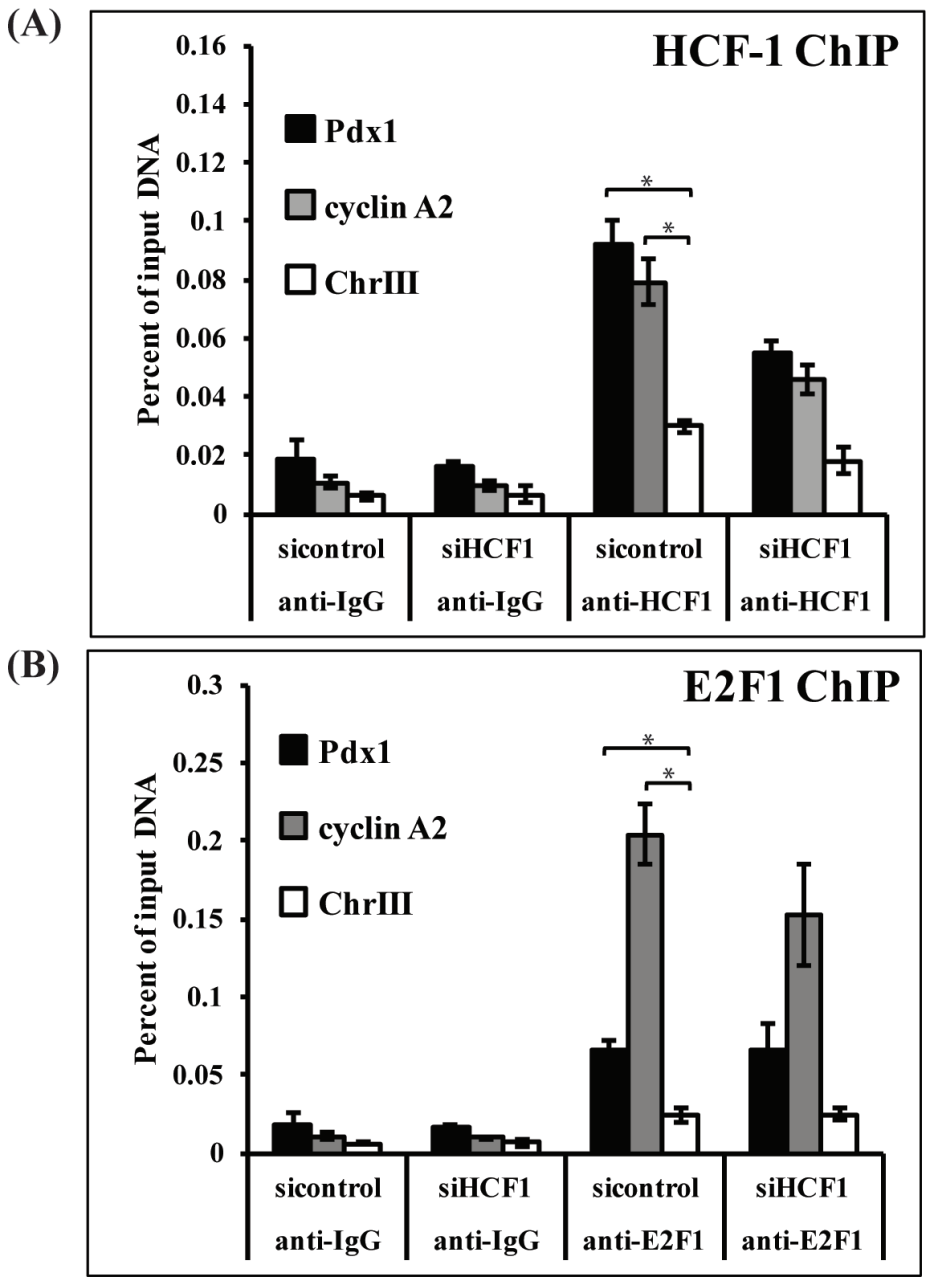
HCF-1 and E2F1 occupy the Pdx1 promoter. INS-1 cells treated with *HCF-1* siRNA or control siRNA were subjected to chromatin immunoprecipitation assays using antibodies directed against (A) HCF-1, (B) E2F1 or isotype-matched rabbit IgG. Immunoprecipitated DNA was quantified using qPCR and calculated as percent of input DNA. Results shown are pooled from three independent experiments and represent the mean +/− SEM. HCF-1 and E2F1 enrichment at the *Pdx1* and *cyclin A2* promoters was significantly greater than at the negative control region (chrIII) (*denotes a p-value <0.05, Student’s t-test). Knockdown of HCF-1 reduced HCF-1 enrichment at the *Pdx1*, *cyclin A2* promoters (p-value = 0.01) to a significantly greater extent than at the control region promoter (p-value = 0.04), whereas E2F1 enrichment was not affected by HCF-1 knockdown. *Cyclin A2* is a known E2F1 target and was included as a control.

Unlike FoxO1, E2F1 localization to the *Pdx1* promoter has not been previously shown. However, E2F1 is known to bind sites predominantly located in the proximal promoter of its target genes, and near to CpG islands [30]. The proximal promoter of the rat *Pdx1* gene contains a highly conserved CpG island [31] as well as a sequence (TTCGCGG) located 168 bp upstream of the transcription start site resembling the consensus E2F1 binding element (TTTXGCGC) [32]. We performed chromatin immunoprecipitation (ChIP) experiments to assess whether HCF-1 and E2F1 bind to the *Pdx1* promoter. Interestingly, we found that HCF-1 enrichment at the *Pdx1* promoter was significantly higher than at the negative control region of chromosome 3, and comparable to that observed at the known HCF-1/E2F1 target gene *cyclin A2* which was included here as a control [12] (Figure 3A). *HCF-1* siRNA treatment reduced HCF-1 occupancy at both the *Pdx1* and *cyclin A2* promoters (Figure 3A). Examination of E2F1 promoter localization also revealed significant enrichment of E2F1 at both the *cyclin A2* promoter as well as at the *Pdx1* promoter, indicating that HCF-1 and E2F1 are both localized to the proximal promoter of *Pdx1* (Figure 3B). E2F1 enrichment, in contrast to HCF-1, was not significantly affected by HCF-1 knockdown (Figure 3B), consistent with previous studies indicating that HCF-1 is not required for E2F1 recruitment to E2F1 target gene promoters [12]. These results indicate that HCF-1 and E2F1 may indeed cooperate to promote *Pdx1* gene regulation. HCF-1 might generally promote E2F1 transcriptional activity in pancreatic β-cells. Consistent with this possibility, we also observe reduced expression of the E2F1 transcriptional target gene *Kir6.2* in HCF-1 knockdown cells (Figure S2).

Our data indicate that HCF-1 modulates the expression of three E2F1 targets (*cyclin A2*, *Pdx1*, and *Kir6*.2), involved in distinct aspects of β-cell function. This may suggest that HCF-1 and E2F1 co-regulation is an important generalized mechanism for maintaining proper β-cell function. Future global analysis to identify additional transcriptional targets commonly regulated by HCF-1 and E2F1 will reveal the key downstream mediators likely contributing to the common phenotypes observed in HCF-1- and E2F1-deficient β-cells. In conclusion, we have identified a role for the transcriptional co-regulator HCF-1 as an essential regulator of *Pdx1* expression, with consequences to β-cell growth and glucose-stimulated insulin secretion. Thus, HCF-1 represents a novel target for future therapies aimed at treating and preventing the progression of diabetes.

## Supporting Information

Figure S1 **HCF-1 is required for proliferation of INS-1 β-cells.**
(TIF)

Figure S2 **HCF-1 knockdown reduces expression of E2F1 target genes.**
(TIF)

Table S1 Sequences of PCR primers used.
(TIF)

## References

[1] DeFronzo RA, Abdul-Ghani MA (2011) Preservation of beta-cell function: the key to diabetes prevention. J Clin Endocrinol Metab 96: 2354–2366. 21697254 10.1210/jc.2011-0246

[2] Mathis D, Vence L, Benoist C (2001) beta-Cell death during progression to diabetes. Nature 414: 792–798. 11742411 10.1038/414792a

[3] Bernardo AS, Hay CW, Docherty K (2008) Pancreatic transcription factors and their role in the birth, life and survival of the pancreatic beta cell. Mol Cell Endocrinol 294: 1–9. 18687378 10.1016/j.mce.2008.07.006

[4] Fu Z, Gilbert ER, Liu D (2013) Regulation of insulin synthesis and secretion and pancreatic Beta-cell dysfunction in diabetes. Curr Diabetes Rev 9: 25–53. 22974359 PMC3934755

[5] Goto H, Motomura S, Wilson AC, Freiman RN, Nakabeppu Y, et al (1997) A single-point mutation in HCF causes temperature-sensitive cell-cycle arrest and disrupts VP16 function. Genes Dev 11: 726–737. 9087427 10.1101/gad.11.6.726

[6] Julien E, Herr W (2003) Proteolytic processing is necessary to separate and ensure proper cell growth and cytokinesis functions of HCF-1. EMBO J 22: 2360–2369. 12743030 10.1093/emboj/cdg242 PMC156000

[7] Tyagi S, Herr W (2009) E2F1 mediates DNA damage and apoptosis through HCF-1 and the MLL family of histone methyltransferases. EMBO J 28: 3185–3195. 19763085 10.1038/emboj.2009.258 PMC2771094

[8] Dejosez M, Krumenacker JS, Zitur LJ, Passeri M, Chu LF, et al (2008) Ronin is essential for embryogenesis and the pluripotency of mouse embryonic stem cells. Cell 133: 1162–1174. 18585351 10.1016/j.cell.2008.05.047 PMC2495776

[9] Wysocka J, Herr W (2003) The herpes simplex virus VP16-induced complex: the makings of a regulatory switch. Trends Biochem Sci 28: 294–304. 12826401 10.1016/S0968-0004(03)00088-4

[10] Wysocka J, Myers MP, Laherty CD, Eisenman RN, Herr W (2003) Human Sin3 deacetylase and trithorax-related Set1/Ash2 histone H3-K4 methyltransferase are tethered together selectively by the cell-proliferation factor HCF-1. Genes Dev 17: 896–911. 12670868 10.1101/gad.252103 PMC196026

[11] Yokoyama A, Wang Z, Wysocka J, Sanyal M, Aufiero DJ, et al (2004) Leukemia proto-oncoprotein MLL forms a SET1-like histone methyltransferase complex with menin to regulate Hox gene expression. Mol Cell Biol 24: 5639–5649. 15199122 10.1128/MCB.24.13.5639-5649.2004 PMC480881

[12] Tyagi S, Chabes AL, Wysocka J, Herr W (2007) E2F activation of S phase promoters via association with HCF-1 and the MLL family of histone H3K4 methyltransferases. Mol Cell 27: 107–119. 17612494 10.1016/j.molcel.2007.05.030

[13] Luciano RL, Wilson AC (2003) HCF-1 functions as a coactivator for the zinc finger protein Krox20. J Biol Chem 278: 51116–51124. 14532282 10.1074/jbc.M303470200 PMC4291123

[14] Ruan HB, Han X, Li MD, Singh JP, Qian K, et al (2012) O-GlcNAc transferase/host cell factor C1 complex regulates gluconeogenesis by modulating PGC-1alpha stability. Cell Metab 16: 226–237. 22883232 10.1016/j.cmet.2012.07.006 PMC3480732

[15] Rizki G, Iwata TN, Li J, Riedel CG, Picard CL, et al (2011) The evolutionarily conserved longevity determinants HCF-1 and SIR-2.1/SIRT1 collaborate to regulate DAF-16/FOXO. PLoS Genet 7: e1002235. 21909281 10.1371/journal.pgen.1002235 PMC3164695

[16] Zmuda EJ, Qi L, Zhu MX, Mirmira RG, Montminy MR, et al (2010) The roles of ATF3, an adaptive-response gene, in high-fat-diet-induced diabetes and pancreatic beta-cell dysfunction. Mol Endocrinol 24: 1423–1433. 20519332 10.1210/me.2009-0463 PMC2903910

[17] Meerbrey KL, Hu G, Kessler JD, Roarty K, Li MZ, et al (2011) The pINDUCER lentiviral toolkit for inducible RNA interference in vitro and in vivo. Proc Natl Acad Sci U S A 108: 3665–3670. 21307310 10.1073/pnas.1019736108 PMC3048138

[18] Ohlsson H, Karlsson K, Edlund T (1993) IPF1, a homeodomain-containing transactivator of the insulin gene. EMBO J 12: 4251–4259. 7901001 10.1002/j.1460-2075.1993.tb06109.x PMC413720

[19] Brissova M, Shiota M, Nicholson WE, Gannon M, Knobel SM, et al (2002) Reduction in pancreatic transcription factor PDX-1 impairs glucose-stimulated insulin secretion. J Biol Chem 277: 11225–11232. 11781323 10.1074/jbc.M111272200

[20] Kulkarni RN, Jhala US, Winnay JN, Krajewski S, Montminy M, et al (2004) PDX-1 haploinsufficiency limits the compensatory islet hyperplasia that occurs in response to insulin resistance. J Clin Invest 114: 828–836. 15372107 10.1172/JCI21845 PMC516265

[21] Wang H, Iezzi M, Theander S, Antinozzi PA, Gauthier BR, et al (2005) Suppression of Pdx-1 perturbs proinsulin processing, insulin secretion and GLP-1 signalling in INS-1 cells. Diabetologia 48: 720–731. 15756539 10.1007/s00125-005-1692-8

[22] Stoffers DA, Ferrer J, Clarke WL, Habener JF (1997) Early-onset type-II diabetes mellitus (MODY4) linked to IPF1. Nat Genet 17: 138–139. 9326926 10.1038/ng1097-138

[23] Stoffers DA, Stanojevic V, Habener JF (1998) Insulin promoter factor-1 gene mutation linked to early-onset type 2 diabetes mellitus directs expression of a dominant negative isoprotein. J Clin Invest 102: 232–241. 9649577 10.1172/JCI2242 PMC509085

[24] Macfarlane WM, Frayling TM, Ellard S, Evans JC, Allen LI, et al (1999) Missense mutations in the insulin promoter factor-1 gene predispose to type 2 diabetes. J Clin Invest 104: R33–39. 10545530 10.1172/JCI7449 PMC481047

[25] Kitamura T, Nakae J, Kitamura Y, Kido Y, Biggs WH 3rd, et al (2002) The forkhead transcription factor Foxo1 links insulin signaling to Pdx1 regulation of pancreatic beta cell growth. J Clin Invest 110: 1839–1847. 12488434 10.1172/JCI200216857 PMC151657

[26] Kawamori D, Kaneto H, Nakatani Y, Matsuoka TA, Matsuhisa M, et al (2006) The forkhead transcription factor Foxo1 bridges the JNK pathway and the transcription factor PDX-1 through its intracellular translocation. J Biol Chem 281: 1091–1098. 16282329 10.1074/jbc.M508510200

[27] Li J, Ebata A, Dong Y, Rizki G, Iwata T, et al (2008) Caenorhabditis elegans HCF-1 functions in longevity maintenance as a DAF-16 regulator. PLoS Biol 6: e233. 18828672 10.1371/journal.pbio.0060233 PMC2553839

[28] Fajas L, Annicotte JS, Miard S, Sarruf D, Watanabe M, et al (2004) Impaired pancreatic growth, beta cell mass, and beta cell function in E2F1 (−/− )mice. J Clin Invest 113: 1288–1295. 15124020 10.1172/JCI18555 PMC398423

[29] Annicotte JS, Blanchet E, Chavey C, Iankova I, Costes S, et al (2009) The CDK4-pRB-E2F1 pathway controls insulin secretion. Nat Cell Biol 11: 1017–1023. 19597485 10.1038/ncb1915 PMC2824657

[30] Bieda M, Xu X, Singer MA, Green R, Farnham PJ (2006) Unbiased location analysis of E2F1-binding sites suggests a widespread role for E2F1 in the human genome. Genome Res 16: 595–605. 16606705 10.1101/gr.4887606 PMC1457046

[31] Park JH, Stoffers DA, Nicholls RD, Simmons RA (2008) Development of type 2 diabetes following intrauterine growth retardation in rats is associated with progressive epigenetic silencing of Pdx1. J Clin Invest 118: 2316–2324. 18464933 10.1172/JCI33655 PMC2373422

[32] Tao Y, Kassatly RF, Cress WD, Horowitz JM (1997) Subunit composition determines E2F DNA-binding site specificity. Mol Cell Biol 17: 6994–7007. 9372931 10.1128/mcb.17.12.6994 PMC232556

