# Supplementary figures and images for: The Transcriptional Co-Regulator HCF-1 Is Required for INS-1 β-cell Glucose-Stimulated Insulin Secretion
Source: PLoS One. 2013 Nov 8;8(11):e78841. doi: 10.1371/journal.pone.0078841 (PMC3826731; doi:10.1371/journal.pone.0078841)

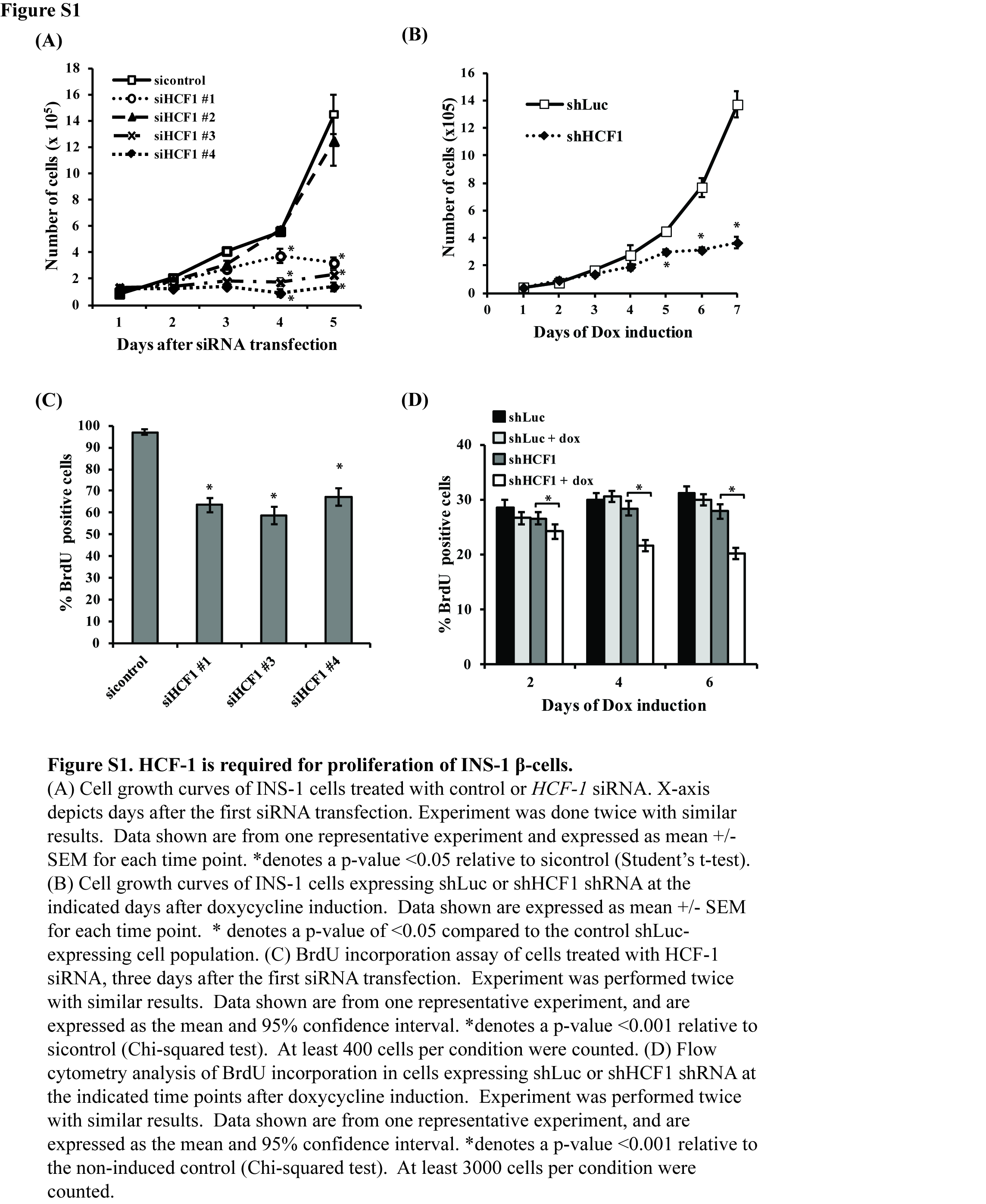

Supplement: Figure S1 — HCF-1 is required for proliferation of INS-1 β-cells. (TIF) [file pone.0078841.s001.tif]

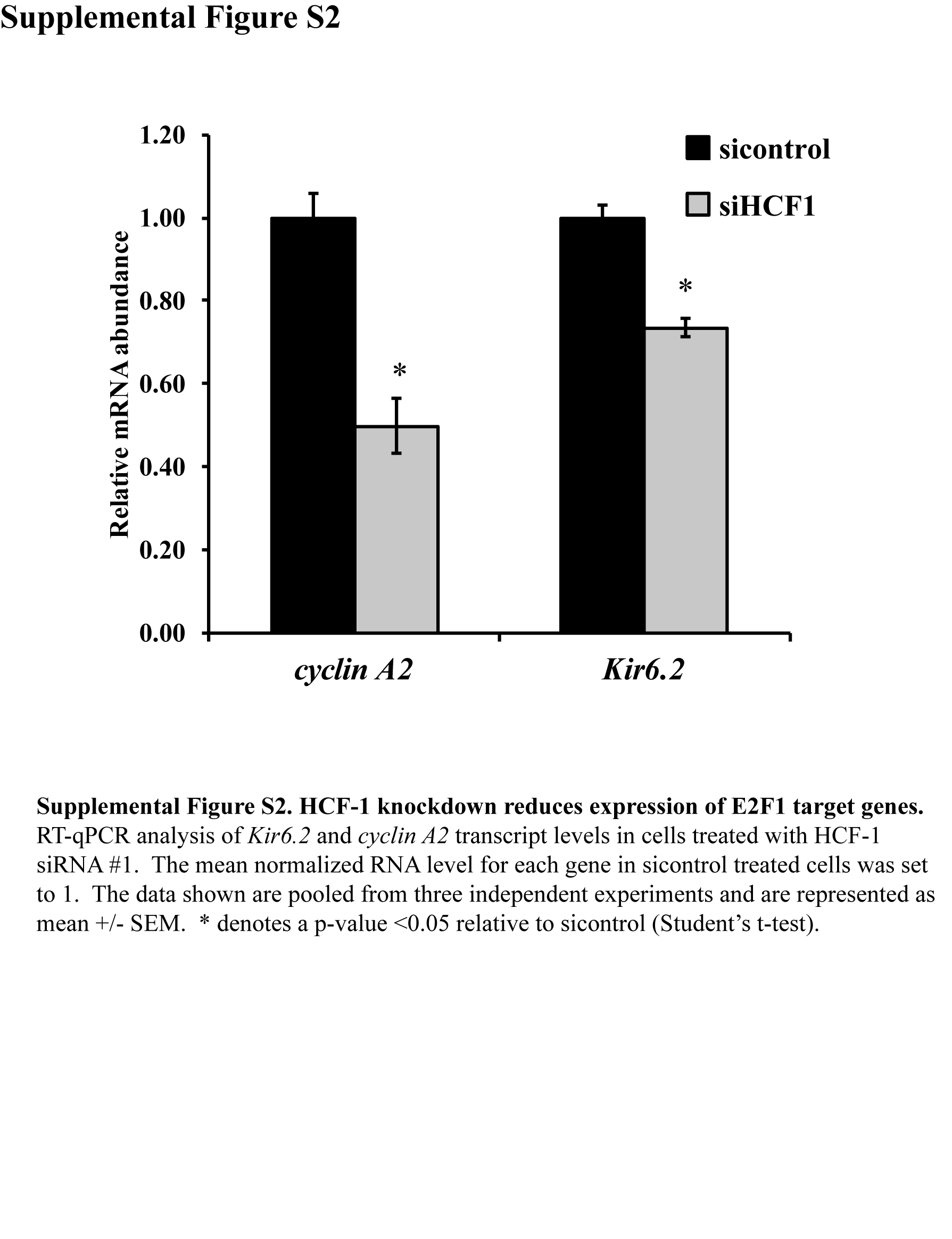

Supplement: Figure S2 — HCF-1 knockdown reduces expression of E2F1 target genes. (TIF) [file pone.0078841.s002.tif]

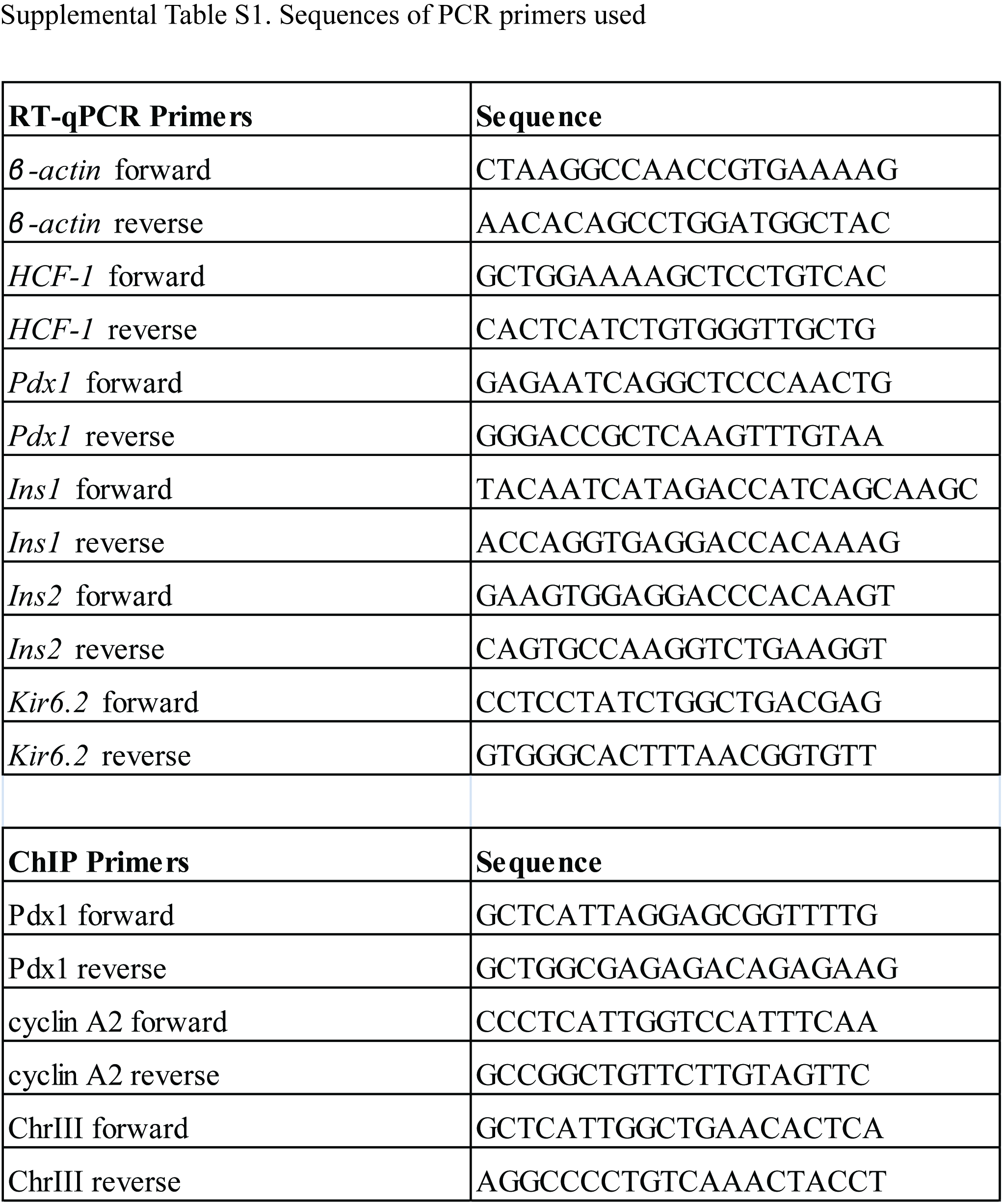

Supplement: Table S1 — Sequences of PCR primers used. (TIF) [file pone.0078841.s003.tif]
